# Y chromosome haplogroups based genome-wide association study pinpoints revelation for interactions on non-obstructive azoospermia

**DOI:** 10.1038/srep33363

**Published:** 2016-09-15

**Authors:** Chuncheng Lu, Yang Wen, Weiyue Hu, Feng Lu, Yufeng Qin, Ying Wang, Shilin Li, Shuping Yang, Yuan Lin, Cheng Wang, Li Jin, Hongbing Shen, Jiahao Sha, Xinru Wang, Zhibin Hu, Yankai Xia

**Affiliations:** 1 State Key Laboratory of Reproductive Medicine, Institute of Toxicology, Nanjing Medical University, Nanjing 210029, China; 2 Key Laboratory of Modern Toxicology of Ministry of Education, School of Public Health, Nanjing Medical University, Nanjing 210029, China; 3 Department of Epidemiology and Biostatistics and Key Laboratory of Modern Toxicology of Ministry of Education, School of Public Health, Nanjing Medical University, Nanjing, China; 4 State Key Laboratory of Genetic Engineering and Ministry of Education Key Laboratory of Contemporary Anthropology, School of Life Sciences, Fudan University, Shanghai 200433, China; 5 Institutes of Biomedical Sciences, Fudan University, Shanghai 200032, China

## Abstract

The Y chromosome has high genetic variability with low rates of parallel and back mutations, which make up the most informative haplotyping system. To examine whether Y chromosome haplogroups (Y-hgs) could modify the effects of autosomal variants on non-obstructive azoospermia (NOA), based on our previous genome-wide association study (GWAS), we conducted a genetic interaction analysis in GWAS subjects. Logistic regression analysis demonstrated a protective effect of Y-hg O3e^*^ on NOA. Then, we explored the potential interaction between Y-hg O3e^*^ and autosomal variants. Our results demonstrated that there was a suggestively significant interaction between Y-hg O3e^*^ and rs11135484 on NOA (*P*_inter_ = 9.89 × 10^−5^). Bioinformatic analysis revealed that genes annotated by significant single nucleotide polymorphisms (SNPs) were mainly enriched in immunological pathways. This is the first study of interactions between Y-hgs and autosomal variants on a genome-wide scale, which addresses the missing heritability in spermatogenic impairment and sheds new light on the pathogenesis of male infertility.

Infertility is the inability of a sexually active couple to achieve pregnancy without contraception in one year^1^. It affects about one in six couples, and male contributions can be found in approximately half of the cases^2^. Apart from some acquired factors like mechanical injury, infection, medical use *et al*., something congenital such as genetic abnormalities plays a crucial role in the etiology of male infertility. Mutagenesis studies *in vivo* have identified hundreds of potential causal genes that impact the process of reproduction^3^,^4^, and some of them, like chromosomal abnormalities and Y chromosome micro-deletions, are used for the diagnosis of male infertility^5^.

Among these genetic studies, Genome-wide association studies (GWAS) present a powerful tool to detect candidate genes for a trait. Our previous GWAS identified some susceptibility loci for non-obstructive azoospermia (NOA) in Han Chinese^6^,^7^. Although GWAS have successfully identified some causal single nucleotide polymorphisms (SNPs) in the past few years, some researchers now find it more limited for complex diseases^8^,^9^. For example, GWAS focus on common SNPs and neglect rare mutations; and SNPs on sex chromosomes are commonly excluded from most GWAS due to the law of linkage disequilibrium. However, sex chromosomes, especially the Y chromosome, play central roles in sex determination, and it is improper to overlook effects of sex chromosomes when understanding the genetic etiology of a disease.

Y chromosome, in contrast to the rest of the genome, is confined to males and contains the smallest number of genes, most of which locate in the male specific region (MSY)^10^. Y chromosome has the most informative haplotyping system with applications in evolutionary studies, medical genetics and genealogical reconstruction^11^. Considering the function of the Y chromosome in sex determination, it has been reported that some Y chromosome haplogroups (Y-hgs) may increase the danger of spermatogenic impairment across different populations^12^,^13^,^14^.

In fact, as a mainly genetic background, Y-hgs may underlie phenotypic variations like SNPs in autosomes. And association studies considering only one of the Y chromosme or autosomes are likely to attain inconsistent results among different human populations or even in the same population^15^,^16^,^17^. A more appropriate strategy to explore potential genetic causes of spermatogenic impairment is to combine Y-hgs with SNPs in autosomes. Therefore, in this study we recruited 1,000 NOA cases and 1,703 fertile controls in Han-Chinese, compared the distributions of Y-hgs in both groups and analyzed the interactions between Y-hgs and autosomal variants in GWAS.

## Results

### Distributions of Y-hgs for NOA

**Figure 1:**
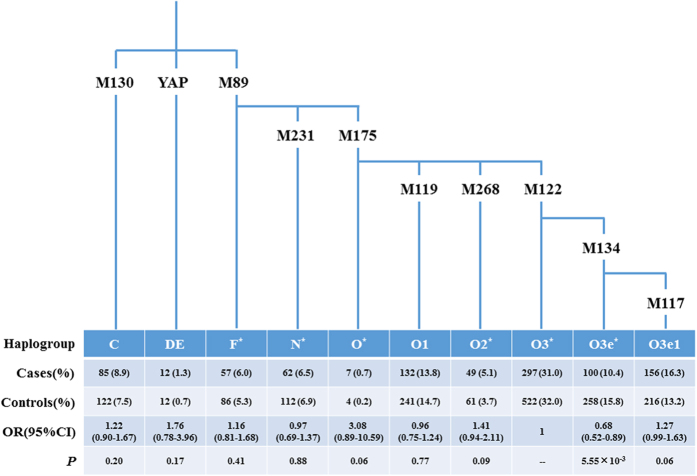
The phylogenetic tree of the Y chromosome and the distribution of the patients and controls in different haplogroups. Each vertical line represents one haplogroup of the Y chromosome phylogenetic tree. Totally 10 common haplogroups are shown and lined up from the older (C, DE, F^*^) on the left to the younger (O3e1) on the right. The upper symbols (M130, YAP, M89, M231, M175, M119, M268, M122, M134 and M117) are markers of the single nucleotide polymorphisms that define different haplogroups according to the Y chromosome Consortium nomenclature. The numbers in the table reflect the number and percentage of cases and controls, odds ratio, 95% confindence intervals and *P* value of each haplogroup in our study.

To assess whether some Y-hgs are predisposing to or protecting against the spermatogenic impairment, we compared the distributions of Y-hgs between cases and controls. As shown in Fig. 1, we found that subjects belonging to Y-hg O3e* were more frequent in controls than in cases, and the difference was statistically significant (OR = 0.68, 95% CI = 0.52–0.89 and *P* = 5.55 × 10^−3^). On the contrary, frequencies of Y-hgs O3e1 and O2* was higher in the case group (16.3% and 5.1%, respectively) than those in the control group (13.2% and 3.7%, respectively), although there were no significant differences between these two groups.

### Interactions between Y-hgs and SNPs on NOA

From the first-step screening, there was only one significant Y-hg O3e* associated with NOA. Thus, in order to examine potential modifications of the Y-hg on effects of SNPs, we further compared the distribution of SNPs among NOA cases and controls based on the frequency of Y-hg O3e*. Although there is no demonstration of a statistically significant result here, however, totally 38 Y-hg O3e*-SNP pairs were found to be suggestively significant (*P*_inter_ ≤ 1 × 10^−4^) and presented in Table 1. Considering the main effects of both Y-hg O3e* and SNPs, rs11135484 possessed the smallest *P* = 9.09 × 10^−4^ and executed a beneficial effect on azoospermia (OR_SNP_ = 0.80). Interaction between Y-hg O3e* and rs11135484 was synergistic (OR_inter_ = 2.07). Besides, rs17217643, rs6774209, rs11135482, rs17139327, rs4757259 and rs8035166 were also protective factors which magnified their effects on the presence of Y-hg O3e*. On the contrary, rs11678378, rs12520985, rs9452333 and rs9510242 showed antagonistic interactions with Y-hg O3e*.

### Further functional study in silicon

**Figure 2:**
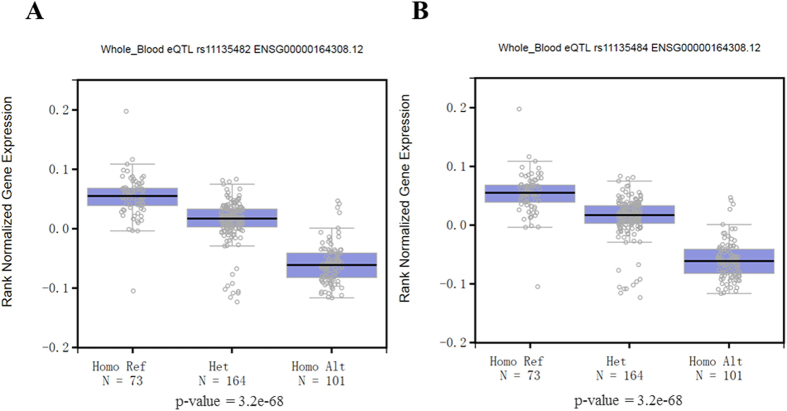
Expression quantitative trait loci (eQTL) box plots of associations between genotypes of rs11135482 (**A**) and rs11135484 (**B**) with *ERAP2* expression from the Genotype-Tissue Expression (GTEx). X-axes are the allele frequencies of two SNPs determined by the hg19/GRCh37 human genome reference. Ref stands for reference allele, and alt stands for alleles that are alternate in comparison to the reference. Y-axes are gene expression obtained from RNA-seq, and rank normalization was performed to bring the expression profile onto the same scale and to protect from outliers. Box plot shows ranked normalized gene expression in median, 1^st^ and 3^rd^ quartiles, 1.5 interquartile range (IQR) of 1^st^ and 3^rd^ quartiles. This plot is from the tissue of whole blood, and both SNPs are also eQTLs of *EARP2* in other tissues including muscle skeletal, lung, adipose subcutaneous, etc.

To examine whether SNPs in Table 1 exert their function via effects on expressions of nearby genes, we searched these variants on the GTEx in all expression quantitative trait loci (eQTL) tissues including blood, lung, adipose etc. As shown in Fig. 2, only two SNPs (rs11135482 and rs11135484) located in chromosome 5 were cis-eQTL. And both of them were significantly associated with a reduced expression of the gene *ERAP2* (*P* = 5.1 × 10^−32^ for both).

In order to explore potential functions of SNPs, we performed pathway analysis using gene ontology (GO) enrichment analysis. As presented in Table 2, 17 biological process pathways were listed. Most of these pathways, including natural killer cell activation, lymphocyte activation, leukocyte activation etc, were belonging to the immunology. The most significant pathway was the Tob1 pathway.

## Discussion

GWAS has become a powerful tool for genetic scientists in the past 10 years. The strategy of GWAS is to uncover the SNPs which occur differently in people with or without a particular disease like cancer, Alzheimer disease, obesity, etc. Using this solution, we have identified several potential risk genomic regions of NOA in our previous studies^6^,^7^. Looking back, however, there is a growing cognition that GWAS approach has its limitations. One of the biggest problems before was whether to choose rare variants of large effect or common variants of small effect, but it is no longer a problem since the development of genotyping technology^18^. Besides that, the neglected sex chromosomes in GWAS are believed to contribute to the “missing” heritability in the etiology of complex diseases.

The X and Y chromosomes, the sex chromosomes, are special for men since the hemizygous exposure. While the abnormalities in the X chromosome are reported to be associated with a much wider range of diseases, the Y chromosome is believed to play a pivotal role in sex determination and spermatogenesis. The deletions of the AZF region in the Y chromosome long arm lead to spermatogenic impairment^19^. Given the haploid nature of the Y chromosome, it is reported to be the major reason for the exclusion in GWAS^20^, and the analysis of Y-hgs that defined by a series of SNPs has been recognized as a more appropriate strategy in the association study^21^. Our efforts using this strategy have proved that some Y-hgs, such as Y-hg K, Q1, are potential risk factors for male infertility^22^,^23^. And there is a great tendency to survey the contribution of sex chromosomes to complex traits for filling the blank space of GWAS^20^.

So in this study, we hypothesized that individuals in predisposed Y-hgs may carry some autosomal variants, which might be a potential genetic modifier for the Y-hg specific susceptibility to spermatogenic impairment. We first examined whether some Y-hgs are risk or protective factors for spermatogenic impairment. Our results demonstrated that Y-hg O3e* was significantly associated with NOA. Compared with the reference Y-hg O3*, Y-hg O3e* was protective against the prevalence of spermatogenic impairment. The diversity of haplogroups is the result of genetic drift, natural selection or stochastic processes, and the beneficial effect on NOA cannot be occasional. Although the machanisms by which haplogroups exert their functions are unclear, emerging evidence has been reported to connect different haplogroups with variations of phenotypes, including high or reduced sperm motility^24^.

Next, to find out the autosomal variants with which Y-hg O3e* might interact, we combined data from Y-hgs with those from our previous GWA study. The pseudo-R^2^, which was a measure of goodness of fit of the statistical model, was also shown. Although pseudo-R^2^ values were somewhat low here, they were still helpful in the model building state as a statistic to evaluate competing models and might be quoteworthy for other researchers on the male infertility. Besides that, we found that rs11135484 was suggestive of an interaction with Y-hg O3e* on NOA. Rs11135484 locates in an intron of *ERAP2*, which was found in the cytoplasm/endoplasmic reticulum (ER) and the plasma membrane. ERAP2 is a proteolytic enzyme site in the ER where it plays a central role in antigen processing and presentation^25^, and it is an attractive candidate involved in immune responses and inflammation^26^. It has been reported that certain SNPs located in *ERAP2* can affect its nonsense-mediated RNA decay and protein expression^25^,^27^. Rs11135484 as well as rs11135482 are proved eQTL of *ERAP2* which means they can regulate the expression of *ERAP2*. Moreover, the Encode project also points out that rs11135484 located in a region marked by enhancer markers, and it may change transcription factors binding affinity resulting in the different expression of *ERAP2*. In addition, a possible effect of ERAP2 deficiency could be an alteration in a quantitative reduction of MHC levels and thus influence the homeostasis of reproductive function^25^.

Pathway enrichment analysis showed that some processes like “natural killer cell activation” and “antigen processing and presentation of peptide antigen via MHC class I” shared common genes involved in immunology. For all we know, immunology is an important biological process that deals with the response of body to exo- or endo- disturbance, and many studies have linked it to the reproductive problems such as infertility, failed *in vivo* fertilization, spontaneous abortions etc^28^,^29^. Y-hg O3e* might regulate these immunological genes to keep the body in a steady state from adverse impacts and maintain a normal reproductive capacity.

In summary, we combined Y-hgs with GWAS to investigate potential interactions between them on NOA, and observed that Y-hg O3e* may modify the risk of some SNPs. These results suggest that both the Y chromosome and autosomes may jointly contribute to the reproductive outcomes. We cannot always divide them into two distinct aspects, and the combination may shed new light on the pathogenesis of male infertility.

## Materials and Methods

### Study population

This study was approved by the ethics review board of Nanjing Medical University, and all experimental protocol were in accordance with guidelines approved by the Institutional Review Board for Human Studies of Nanjing Medical University. Totally, 1,000 NOA cases and 1,703 male controls included in this study were reported in our previous genome-wide association study^6^, and a written informed consent was obtained from each subject. Briefly, all infertile cases were genetically unrelated Han Chinese men diagnosed to have idiopathic male infertility without a history of cryptorchidism, vascular trauma, orchitis, obstruction of the vas deferens, vasectomy, chromosome abnormalities or Y chromosome microdeletions of azoospermia factor (AZF) region. Semen analysis was performed following World Health Organization (WHO) criteria (2010)^30^. To ensure the accuracy of the diagnosis, each sample was examined twice. And the absence of spermatozoa in both replicate samples was defined as azoospermia. Fertile control subjects who had fathered one or more healthy children without performing assisted reproductive technology (ART) were shared with the Nanjing Lung Cancer Study.

### Y chromosome haplotyping

Y chromosome haplogtyping was performed in all 2703 participants. Totally, 10 highly informative polymorphic loci for East Asians (M130, YAP, M89, M231, M175, M119, M268, M122, M134 and M117, which defined Y-hgs C, DE, F*, N*, O*, O1, O2*, O3*, O3e*, O3e1) were identified following the nomenclature recommended by the Y Chromosome Consortium (YCC) and its updates^31^,^32^,^33^. The experimental procedures, mainly involving multiplex PCR amplification, restriction fragment length polymorphism (RFLP) and capillary electrophoresis, were described previously^14^.

### Genotyping and quality control in GWAS

The GWAS was conducted using an Affymetrix Genome-Wide Human SNP Array 6.0 followed by a quality control procedure as described previously^6^. In brief, SNPs were excluded if they: did not map to autosomes, had a call rate of <95%, had a minor allele frequency <0.05 or had a genotype distribution in the controls that deviated from that expected with Hardy-Weinberg equilibrium (*P* < 1 × 10^−5^). Individuals with overall genotype completion rates <95%, gender discrepancies, unexpected duplicates or probable relatives, heterozygosity rates >6 s.d. away from the mean or outliers in the principal component analysis were also excluded. After quality control processing, a total of 957 NOA cases and 1634 healthy controls with 587347 SNPs were included in the subsequent interaction analysis.

### Statistical analyses

Statistical analyses were performed using R software (version 3.1.2; The R Foundation for Statistical Computing). Firstly, distributions of Y-hg among cases and controls were assessed, and the statistically significant Y-hg was further combined with SNPs. For the analysis of the Y-hg × SNP interaction, we firstly defined the Y-hg variable as Y-hg O3e* (1) or non-Y-hg O3e* (0). And SNPs were coded as continuous variables (0, 1 and 2) under an additive genetic model. Then we tested the interaction between each pair of Y-hg and SNP by conducting a 1-degree-of-freedom Wald test of a single interaction term as implemented in an unconditional logistic regression based on the equation Y = β_0_ + β_1_ × Y-hg + β_2_ × SNP + β_3_ × (Y-hg × SNP). Here, Y is the logit of the probability of being an infertile case, β_0_ is a constant, β_1_ and β_2_ are the main effects of Y-hg and SNP, respectively, and β_3_ is the interaction parameter to be tested. *P* value ≤ 1 × 10^−8^ was regarded as statistically significant in interaction analyses considering the issue of multiple comparison, and *P* value ≤ 1 × 10^−4^ was suggestive of an interaction.

### Gene ontology (GO) and expression quantitative trait loci (eQTL) analysis

GO enrichment analysis (http://geneontology.org/) was performed to examine biological processes encompassing statistically significant genes involved in our study. In addition, using publicly available data from the Genotype-Tissue Expression (GTEx) eQTL Browser (http://www.gtexportal.org/home/), we examined *cis* associations between SNPs and expression of nearby genes in different tissues.

## Additional Information

**How to cite this article**: Lu, C. *et al*. Y chromosome haplogroups based genome-wide association study pinpoints revelation for interactions on non-obstructive azoospermia. *Sci. Rep.* **6**, 33363; doi: 10.1038/srep33363 (2016).

## Figures and Tables

**Table 1:** Suggestively significant interactions between Y-hg O3e* and SNPs in control and case groups.

Chr	SNP^a^	MAF^b^	MAF^c^	OR_SNP_	*P*_SNP_	OR_O3e*_	*P*_O3e*_	OR_inter_	*P*_inter_	R^*2*^
2	rs13017562	0.38	0.36	0.93	0.26	0.31	3.63 × 10^−8^	2.22	2.62 × 10^−5^	0.064
2	rs869633	0.38	0.36	0.93	0.26	0.31	3.72 × 10^−8^	2.22	2.69 × 10^−5^	0.064
2	rs869632	0.38	0.36	0.94	0.29	0.32	4.69 × 10^−8^	2.18	3.68 × 10^−5^	0.063
2	rs815808	0.06	0.09	0.91	0.37	0.45	1.10 × 10^−7^	3.42	3.05 × 10^−5^	0.065
2	rs815807	0.05	0.08	0.91	0.40	0.46	2.08 × 10^−7^	3.36	6.83 × 10^−5^	0.063
2	rs1723493	0.04	0.08	0.91	0.45	0.47	2.28 × 10^−7^	3.34	9.03 × 10^−5^	0.063
2	rs11678378	0.44	0.43	1.00	0.99	1.12	0.560	0.46	7.63 × 10^−5^	0.063
3	rs17217643	0.31	0.33	0.87	0.03	0.35	5.42 × 10^−8^	2.26	5.25 × 10^−5^	0.063
3	rs6774209	0.31	0.33	0.87	0.03	0.34	4.45 × 10^−8^	2.24	9.16 × 10^−5^	0.065
3	rs10513814	0.19	0.20	0.86	0.07	0.41	1.08 × 10^−7^	2.30	9.75 × 10^−5^	0.062
4	rs17005650	0.33	0.37	1.05	0.44	0.32	5.99 × 10^−8^	2.26	3.47 × 10^−5^	0.066
5	rs11135482	0.39	0.42	0.83	4.93 × 10^−3^	0.31	1.02 × 10^−7^	2.07	9.11 × 10^−5^	0.064
5	rs11135484	0.39	0.43	0.80	9.09 × 10^−4^	0.31	1.56 × 10^−7^	2.07	9.89 × 10^−5^	0.065
5	rs12520985	0.23	0.24	1.11	0.14	0.89	0.455	0.38	7.15 × 10^−5^	0.064
5	rs13181162	0.41	0.41	0.94	0.31	0.30	1.36 × 10^−7^	2.10	9.36 × 10^−5^	0.062
6	rs16891417	0.09	0.06	0.91	0.44	0.49	5.41 × 10^−7^	4.52	7.07 × 10^−5^	0.063
6	rs3818947	0.08	0.06	0.89	0.38	0.49	5.54 × 10^−7^	4.58	6.26 × 10^−5^	0.063
6	rs16891497	0.09	0.06	0.95	0.69	0.48	3.37 × 10^−7^	4.45	4.41 × 10^−5^	0.063
6	rs16891501	0.09	0.06	0.86	0.25	0.49	5.48 × 10^−7^	4.23	7.95 × 10^−5^	0.062
6	rs9452333	0.34	0.42	1.02	0.77	1.04	0.847	0.47	9.88 × 10^−5^	0.063
7	rs17139327	0.22	0.25	0.85	0.03	0.39	5.83 × 10^−8^	2.42	6.11 × 10^−5^	0.064
8	rs10957317	0.15	0.15	0.86	0.08	0.42	5.78 × 10^−8^	2.79	3.05 × 10^−5^	0.063
8	rs12545097	0.15	0.15	0.86	0.09	0.41	3.63 × 10^−8^	2.95	1.20 × 10^−5^	0.064
8	rs2385127	0.15	0.15	0.85	0.07	0.42	4.95 × 10^−8^	2.88	1.92 × 10^−5^	0.064
9	rs2117103	0.18	0.19	0.93	0.34	0.40	6.98 × 10^−8^	2.37	6.17 × 10^−5^	0.063
9	rs1368677	0.18	0.19	0.91	0.26	0.40	6.65 × 10^−8^	2.40	4.77 × 10^−5^	0.063
9	rs12555036	0.42	0.44	0.94	0.29	0.29	1.36 × 10^−7^	2.08	8.48 × 10^−5^	0.063
11	rs4757259	0.30	0.26	0.86	0.04	0.38	6.48 × 10^−8^	2.40	7.45 × 10^−5^	0.063
11	rs4757260	0.30	0.26	0.87	0.06	0.38	6.01 × 10^−8^	2.43	5.82 × 10^−5^	0.063
11	rs12286075	0.32	0.36	0.93	0.28	0.32	5.98 × 10^−8^	2.20	4.92 × 10^−5^	0.063
11	rs4923182	0.42	0.46	0.98	0.70	0.24	1.12 × 10^−8^	2.51	4.12 × 10^−6^	0.067
11	rs4923186	0.42	0.46	0.99	0.85	0.24	1.83 × 10^−8^	2.50	5.04 × 10^−6^	0.066
12	rs10841420	0.44	0.46	0.89	0.07	0.29	5.66 × 10^−8^	2.13	5.78 × 10^−5^	0.064
12	rs10437774	0.43	0.44	0.90	0.09	0.31	1.05 × 10^−7^	2.09	8.46 × 10^−5^	0.063
13	rs9510242	0.33	0.31	1.08	0.23	0.96	0.790	0.43	8.02 × 10^−5^	0.063
15	rs16968382	0.03	0.05	0.81	0.15	0.50	6.47 × 10^−7^	4.09	9.73 × 10^−5^	0.062
15	rs8035166	0.36	0.35	0.85	0.01	0.34	9.47 × 10^−8^	2.15	8.29 × 10^−5^	0.063
20	rs12625552	0.03	0.06	0.85	0.23	0.47	2.19 × 10^−7^	3.99	2.87 × 10^−5^	0.064

OR for odds ratio, *P* for *P* value and R^2^ for goodness of fit of logistic regression models.

^a^Only those suggestively significant SNPs with a *P*_inter_ < 1 × 10^−4^ were shown.

^b^MAF for minor allele frequency in Chinese in 1000 Genomes, ^c^MAF in this study controls.

**Table 2:** Gene Ontology (GO) pathway analysis of Y-hg O3e^*^ interacted SNPs.

Index	Pathway enrichment	*P*	*P*_FDR_
1	Tob1 Pathway	<0.001	<0.001
2	Hematopoietin interferon classd200 domain cytokine receptor binding (GO:0005126)	<0.001	<0.001
3	Homeostasis of number of cells (GO:0048872)	0.001	0.003
4	Natural killer cell activation (GO:0030101)	<0.001	0.003
5	IL18 Pathway	<0.001	0.003
6	Lymphocyte activation (GO:0046649)	<0.001	0.006
7	Cell activation (GO:0001775)	0.001	0.007
8	Antigen processing and presentation of endogenous peptide antigen (GO:0002483)	0.001	0.007
9	Positive regulation of mononuclear cell proliferation (GO:0032946)	0.001	0.008
10	Lymphocyte proliferation (GO:0046651)	<0.001	0.008
11	Leukocyte activation (GO:0045321)	0.001	0.009
12	Regulation of cell growth (GO:0001558)	0.006	0.023
13	Antigen processing and presentation of peptide antigen via MHC class i (GO:0002474)	0.005	0.025
14	Regulation of mononuclear cell proliferation (GO:0032944)	0.007	0.031
15	T cell homeostasis (GO:0043029)	0.017	0.032
16	Regulation of blood pressure (GO:0008217)	0.010	0.034
17	Lymphocyte mediated immunity (GO:0002449)	0.017	0.044
